# Artery of Percheron infarction presenting as nuclear third nerve palsy and transient loss of consciousness: a case report

**DOI:** 10.1186/s12883-020-01889-9

**Published:** 2020-08-28

**Authors:** K. M. I. U. Ranasinghe, H. M. M. T. B. Herath, D. Dissanayake, M. Seneviratne

**Affiliations:** National Hospital of Sri Lanka, Colombo, Sri Lanka

**Keywords:** Artery of Percheron (AOP), Case report, Paramedian thalamic infarction, Thalamic infarction, Mid brain infarction, Nuclear third nerve palsy

## Abstract

**Background:**

Thalamic blood supply consists of four major vascular territories. Out of them paramedian arteries supply ipsilateral paramedian thalami and occasionally rostral mid brain. Rarely both paramedian arteries arise from a common trunk that arise from P1 segment of one sided posterior cerebral artery (PCA). This is usually due to hypoplastic or absent other P1 and this common trunk is termed Artery of Percheron (AOP). Its prevalence is in the range of 7–11% among the general population and AOP infarcts account in an average of 0.4–0.5% of ischemic strokes. Clinical presentation of AOP infarction is characterized by impaired arousal and memory, language impairment and vertical gaze palsy. It also can present with cerebellar signs, hemi paresis and hemi sensory loss. We herein present a case of AOP infarction presenting as transient loss of consciousness and nuclear third nerve palsy.

**Case presentation:**

A 51 year old previously healthy male, was brought to us, with a Glasgow coma scale (GCS) of 7/15. GCS improved to 11/15 by the next day, however he had a persisting expressive aphasia. Right sided nuclear third nerve palsy was apparent with the improvement of GCS. He did not have pyramidal or cerebellar signs. Thrombolysis was not offered as the therapeutic window was exceeded by the time of diagnosis. Diagnosis was made using magnetic resonance imaging (MRI) that was done after the initial normal non-contrast computer tomography (NCCT) brain. He was enrolled in stroke rehabilitation. Aspirin and atorvastatin was started for the secondary prevention of stroke. He achieved independency of advanced daily living by 1 month, however could not achieve full recovery to be employed as a taxi driver.

**Conclusions:**

Because of the rarity and varied clinical presentation with altered levels of consciousness, AOP infarcts are easily overlooked as a stroke leading to delayed diagnosis. Timely diagnosis can prevent unnecessary investigations and the patient will be benefitted by early revascularization. As it is seldom reported, case reports remain a valuable source of improving awareness among physicians about this clinical entity.

## Background

Artery of Percheron (AOP) is an anatomical variant of posterior circulation arteries of the brain. Gerald Percheron studied thalamic blood supply and described its anatomical variants depending on the arteries it arise [1]. According to him, P1 segment of the PCA gives rise to paramedian arteries of each side to supply paramedian thalami. Sometimes these paramedian arteries can arise from one common trunk arising from a P1 segment of either side. This is usually due to hypoplastic or absent other P1 segment. This common trunk is known as AOP. A compromise of the blood flow of AOP leads to bilateral paramedian thalamic infarction with or without rostral mid brain involvement, resulting in characteristic clinical picture of impaired arousal and memory, language impairment and occasionally ocular movement disorders [2–4]. AOP infarction is only seldom associated with pyramidal symptoms. Non contrast computer tomography (NCCT) of the brain is often normal misleading the physician to consider pathologies other than a stroke.

The exact prevalence of AOP remains unknown. But according to two autopsy studies, this anatomical variant can be present in 7 to 11.7% [5, 6]. Though it seems to be a common anatomical variant, strokes due to AOP territory infarction are rare. Its incidence was 0.4% in an analysis done in Mexico involving 3750 patients with first ever ischemic stroke [3] and was 0.5% in another analysis on 3712 patients with ischemic stroke [4]. Out of thalamic strokes, AOP territory strokes lie in the range of 4–18% [7, 8]. Its rarity and the unusual clinical presentation for a stroke, cause a delay in institution of advanced brain imaging and diagnosis. Thus, a good knowledge of the clinical presentation of this distinct entity will be helpful in preventing undue delay in the diagnosis and the administration of thrombolytic therapy when indicated.

## Case presentation

A 51-year-old previously healthy male was brought by his family members following sudden loss of consciousness. On admission his Glasgow coma scale (GCS) was 7/15 (eye 1, verbal 2, and motor 4). Neurological examination revealed bilateral symmetrical sluggish pupils of 3 mm. We could do only a limited neurological examination due to low GCS. He moved all four limbs to a painful stimulus and the deep tendon reflexes were normal. Bilateral flexor plantar response was present. With the suspicion of any drug overdose, a urine sample for toxins was sent urgently, but all the tested toxins including opioids, benzodiazepines and amphetamines were negative. An urgent NCCT brain was done within two hours of symptom onset to exclude intracranial hemorrhage and it turned out to be normal.

**Fig. 1:**
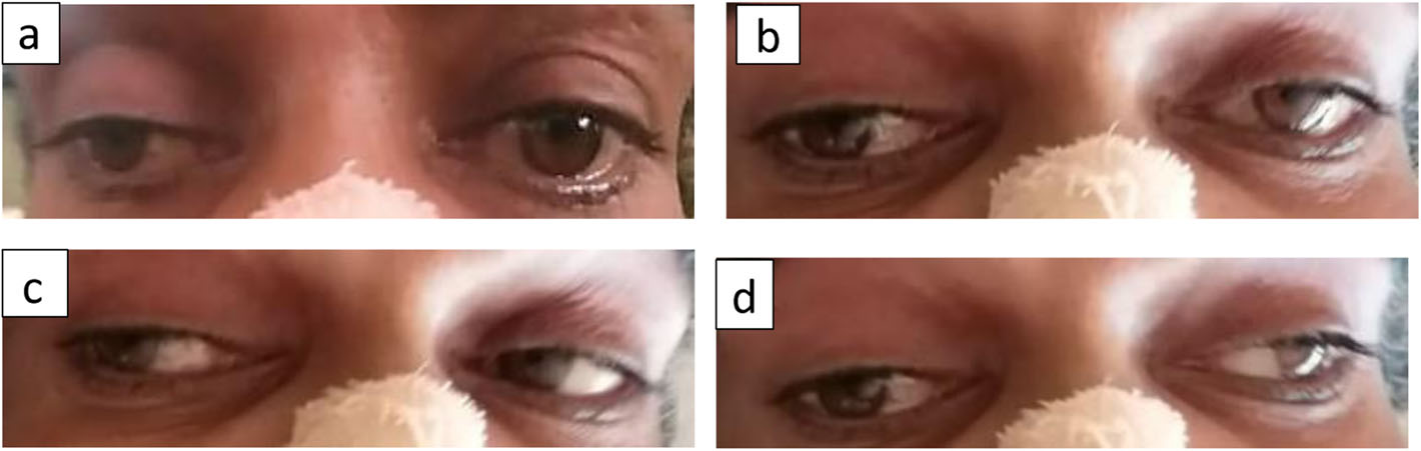
Eye movements demonstrating right eye nuclear third nerve palsy. **a** Neutral position of eyes showing bilateral partial ptosis (right > left) and outward and downward deviation of the right eye due to third nerve palsy. **b** Attempted vertical gaze; vertical gaze palsy is apparent. **c** Right gaze; right eye abduction and left eye adduction is preserved. **d** Left gaze; right eye adduction is impaired whereas left eye abduction is preserved

After four hours of admission, his GCS improved to 11/15 (E3V2M6). However he was drowsy and there was bilateral asymmetrical ptosis, right more than left. Third nerve palsy without pupillary involvement was evident on the right side. Right eye medial and downward gaze were impaired. He also had bilateral upward gaze palsy, but had no nystagmus (Fig. 1). These findings suggested a right-sided nuclear third nerve palsy. He did not have pyramidal or cerebellar signs and the visual fields were normal.

**Fig. 2:**
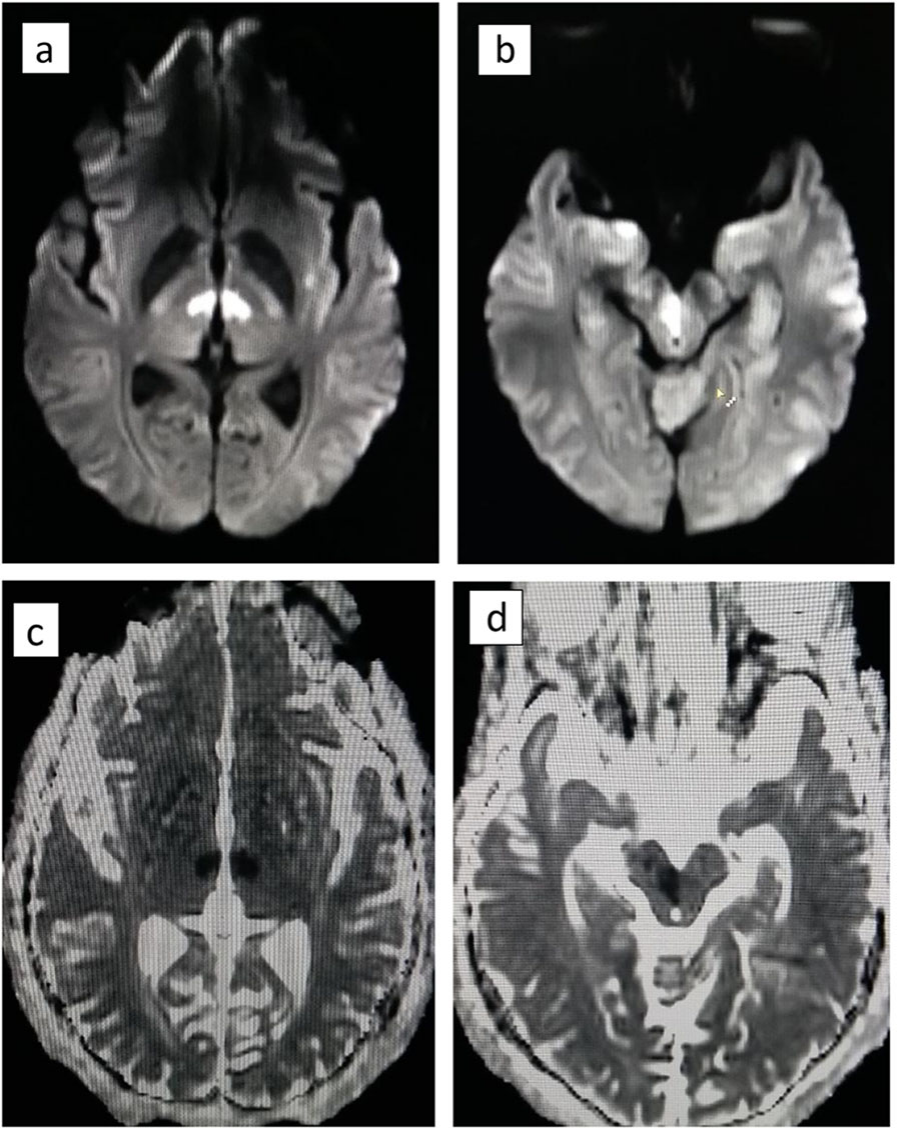
MRI images showing AOP territory infarction. **a** DWI image showing paramedian thalamic infarctions. **b** DWI image showing right rostral midbrain infarction (Note the assymetry in rostral mid brain involvement). **c** ADC image showing paramedian thalamic infarctions. **d** ADC image showing assymetrical right rostral midbrain infarction

As the NCCT brain was normal, we proceeded with magnetic resonance imaging (MRI) and magnetic resonance angiogram (MRA) brain. It was reported as acute infarction in bilateral paramedian thalami and medial rostral mid brain, suggesting AOP territory infarction. MRA showed hypoplastic right vertebral artery. Apparent diffusion coefficient (ADC) images and diffusion weighted images (DWI) showed the paramedian thalamic infarctions in the index case due to AOP involvement. Here the rostral mid brain involvement is asymmetrical as was suggested by the clinical findings as well (Fig. 2).

Over a week, his hypersomnolance gradually improved but his diplopia persisted. After the diagnosis of ischemic stroke, the underlying risk factor assessment was performed. He had hypertension, but no diabetes mellitus. Lipid profile was deranged with a low density lipoprotein of 140 mg/dL. Electrocardiogram did not show any evidence of arrhythmia or past cardiac ischemic events. Transthoracic echocardiogram was normal except for the left ventricular hypertrophy. He did not have polycythemia or thrombocytosis. As he had conventional risk factors for a stroke, we did not further evaluate for other thrombophilic conditions. He was discharged after 1 week of hospitalization with out-patient speech and physiotherapy. We started aspirin 75 mg, clopidogrel 75 mg and atorvastatin 40 mg daily. At the clinic visit scheduled 3 weeks after the discharge, clopidogrel was withheld and aspirin 75 mg and atorvastatin 40 mg were continued. By the time of clinic visit he had a marked recovery and could engage in his day-to-day activities. However he could not resume his work as a taxi driver even 3 months after the initial stroke.

## Discussion and conclusions

Thalamus consists of five major functional nuclei i.e. nociception and arousal controlling intralaminar and reticular nuclei, sensory nuclei receiving inputs from all major sensory domains, effector nuclei controlling language and motor function, associative nuclei that involve in higher cognitive functions and limbic nuclei associated with motivation and mood [9]. These nuclei get their blood supply primarily from the posterior cerebral circulation via different perforating arteries.

**Fig. 3:**
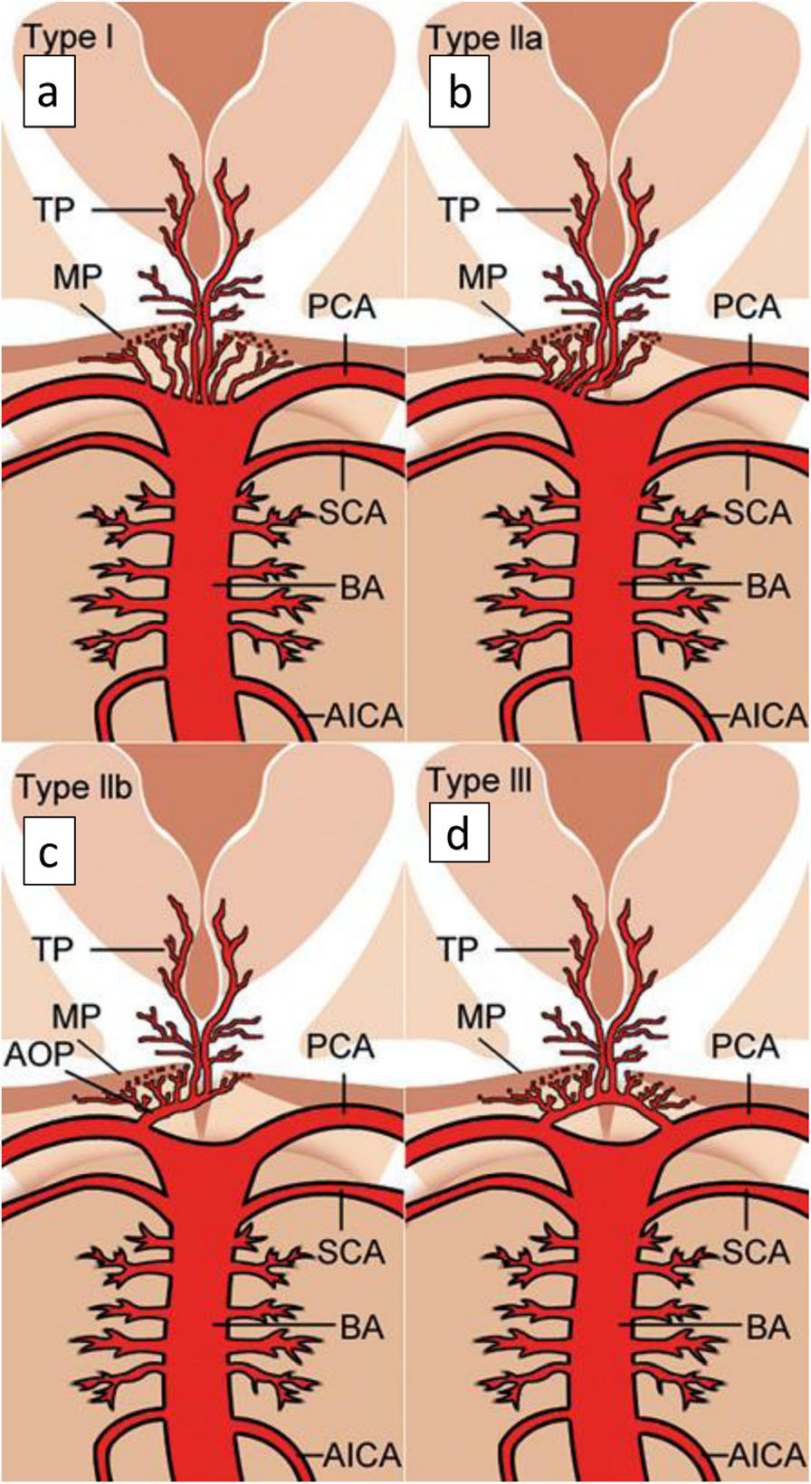
Neurovascular anatomical variants supplying paramedian thalamus and mid brain as was described by Percheron [10]. (Adapted from, Artery of Percheron infarction: review of literature with a case report. Radiology and oncology. 2015 Jun 1;49 (2):141–6.Written permission was obtained from the author and the journal to use as an open access figure). **a** Variant I - each side perforating arteries arising from each PCA. **b** Variant IIa - asymmetrically arising two perforating arteries from one sided PCA. **c** Variant IIb - bilateral thalamic perforating arteries arising from a single common trunk (AOP). **d** Variant III - an arc bridging P1 segments of bilateral PCA giving rise to several perforating arteries. Vessels marked by initials: AICA - anterior inferior cerebellar artery, AOP - artery of Percheron, BA - basilar artery, MP - midbrain perforators, PCA – posterior cerebral artery, SCA - superior cerebellar artery, TP - thalamic perforators

As described by Percheron, thalamic circulation has four variations of arterial supply. The following figure (Fig. 3) demonstrates neurovascular anatomical variants supplying thalamus and mid brain. Out of the four variants, variant I is the most common type and in that each side perforating arteries arise from each PCA. In variant IIa, asymmetrically arising two perforating arteries from one sided PCA supply the thalamus. Variant IIb is the AOP of interest where bilateral thalamic perforating arteries arise from a single common trunk. That common trunk is named AOP [1, 10]. It supplies the paramedian thalami of both sides along with the rostral mid brain [10] Variant III is an arc bridging P1 segments of bilateral PCA giving rise to several perforating arteries that supply the thalamus.

Vascular compromise to the thalamus can lead to various clinical pictures depending on the territory infarcted. In the literature, four major vascular territories of thalamus are described with different clinical presentations of relevant vascular compromise [2]. They are as follows.

i. Tubulothalamic territory – causes impairment in orientation, arousal, memory, learning, personality and executive function
ii. Inferolateral territory – causes hemi sensory loss, hemi paresis, hemi ataxia of the contralateral side and post stroke painful syndrome, especially with right sided strokes
iii. Posteror choroidal territory – causes sensory loss of the contralateral side, visual field defects, tremor, dystonia, weakness and occasional language impairment
iv. Paramedian territory – causes hypersomnolance if the lesion is bilateral, memory impairment, difficulty in learning and language deficits

The artery supplying the tubulothalamic territory can be rarely absent, in which case artery supplying the paramedian territory takes over to supply the former territory as well. In such situations when the AOP variant also coexists, AOP territory infarction can be devastating as it will involve both the aforementioned territories [11].

AOP territory strokes are limited to case reports or small case series in the literature. All of them had an involvement of bilateral paramedian thalami. In addition, polar thalamic nuclei and mid brain were involved in some cases [8, 12, 13]. In a case series of fifteen patients with AOP infarctions, which is the largest case series in the literature to the best of our knowledge, there were three patterns of infarction described [4]. They are,

i. Bilateral paramedian thalamic and rostral mid brain infarctions – 8 patients out of 15 (53%) had this pattern. The main presentations were mental state deficits, ocular movement deficit, aphasia or dysarthria and amnesia. Prognosis was poor in this group.
ii. Bilateral paramedian thalamic infarctions without midbrain involvement – 6 patients (40%) were in this group. The main presentations were mental state deficits and amnesia. In addition, cerebellar signs were seen in 50%. Ocular movement deficits were less common, noted only in two patients (33%). Prognosis was favorable.
iii. Bilateral paramedian and anterior thalamic infarctions without mid brain involvement – Only one patient out of the fifteen had this presentation. The reported symptoms and signs were mental state deficits, ocular movement disorder and aphasia. Prognosis was favorable.

Our patient had the first presentation described above with bilateral paramedian and rostral mid brain infarctions. As was seen in the above case series, he presented with an impaired mental status, with a GCS of 7/15. He had global aphasia initially. Ocular movement deficits he had were right sided nuclear third nerve palsy and bilateral upward gaze palsy. Partial ptosis was asymmetrical with the more affected right side. He did not have cerebellar or pyramidal signs.

The third nerve nucleus is a complex of sub nuclei. Within the complex, sub nucleus to levator palpebral superioris is unpaired. Thus a lesion at the nuclear level will cause bilateral partial ptosis [14]. Furthermore the superior rectus muscle has a crossed innervation whereas motor neurons to medial rectus, inferior rectus and inferior oblique are uncrossed. So in case of nuclear third nerve palsy, there will be bilateral superior rectus palsy due to the involvement of ipsilateral superior rectus nucleus and the crossing fibers from the other side [15].

Our patient’s MRI and MRA findings suggested AOP territory involvement in which a symmetrical neurological deficit is usually expected. AOP was not visualized in MRA imaging of our patient probably due to its occlusion. Its anatomical asymmetry in our patient would explain the right sided third nerve involvement. In case of AOP infarct, the initial NCCT brain remains normal most of the time, whereas the diagnosis is made with a subsequent MRI and diffusion weighted imaging (DWI). AOP is seldom demonstrated by imaging as it is too small to be visualized by MRA or CT angiogram, or as it is occluded [16, 17].

The underlying etiologies for AOP territory infarction remain the usual causes like hypertension, dyslipidemia, diabetes, smoking, atrial fibrillation etc. as for any other ischemic stroke. In a case series of 10 patients with AOP territory strokes, 60% had small artery disease whereas 40% had cardiac source of embolism [2]. Our patient was diagnosed with hypertension and dyslipidemia. He did not have diabetes and echocardiogram was normal. So we attributed small artery disease as the potential cause of his stroke.

If the timely diagnosis of AOP infarct is made within the therapeutic window of 4.5 h, available treatment modalities include intravenous thrombolysis and endovascular treatment [18]. But mostly due to its varied presentation, diagnosis is made outside the therapeutic window, as happened with our patient as well. Prognosis of thalamic strokes is usually favorable, which applies to AOP territory infarctions as well [2, 4, 9]. In the aforementioned case series of 15 patients, 67% of who had bilateral paramedian thalamic infarcts had a functional independency with a Modified Rankin Scale score less than 2. However those who had rostral midbrain involvement in addition to the paramedian thalamic infarctions, had a poorer outcome. Only 25% of them had a favorable outcome [4]. Our patient’s hypersomnolance improved by day seven and at the 3 week review he was independent in his day to day activities. However he did not recover fully to resume his work as a taxi driver even after 3 months.

As discussed above, AOP infarctions remain a rare subset of ischemic strokes and are commonly overlooked due to their relative lack of well-known motor deficits that are expected in a stroke. Timely diagnosis can prevent unnecessary investigations and also the patient will be benefitted if revascularization is achieved by timely therapeutic interventions. Because of the rarity of AOP infarctions, case reports remain to be a valuable source of improving awareness amongst physicians.

## Data Availability

All necessary data and material are provided.

## Abbreviations

ADC: Afferent diffusion coefficient
AOP: Artery of Percheron
DWI: Diffusion weighed imaging
GCS: Glasgow coma scale
MRI: Magnetic resonance imaging
MRA: Magnetic resonance angiography
NCCT: Non contrast computer tomography
PCA: Posterior cerebral artery
